# A Short Promoter Region Containing Conserved Regulatory Motifs Is Required for Steroidogenic Acute Regulatory Protein (*Star*) Gene Expression in the Mouse Testis

**DOI:** 10.3390/ijms231912009

**Published:** 2022-10-09

**Authors:** Marie France Bouchard, Julia Picard, Jacques J. Tremblay, Robert S. Viger

**Affiliations:** 1 Reproduction, Mother and Child Health, Centre de Recherche du CHU de Québec-Université Laval and Centre de Recherche en Reproduction, Développement et Santé Intergénérationnelle (CRDSI), Québec, QC GIV4G2, Canada; 2 Department of Obstetrics, Gynecology, and Reproduction, Université Laval, Québec, QC G1K7P4, Canada

**Keywords:** steroidogenesis, testis, transcription, testosterone, Leydig cell, GATA

## Abstract

In the testis, Leydig cells produce steroid hormones that are needed to masculinize typical genetic males during fetal development and to initiate and maintain spermatogenesis at puberty and adulthood, respectively. Steroidogenesis is initiated by the transfer of cholesterol from the outer to the inner mitochondrial membrane through the action of steroidogenic acute regulatory protein (STAR). Given its importance for the steroidogenic process, the regulation of *STAR* gene expression has been the subject of numerous studies. These studies have involved the characterization of key promoter sequences through the identification of relevant transcription factors and the nucleotide motifs (regulatory elements) that they bind. This work has traditionally relied on in vitro studies carried out in cell cultures along with reconstructed promoter sequences. While this approach has been useful for developing models of how a gene might be transcriptionally regulated, one must ultimately validate that these modes of regulation occur in an endogenous context. We have used CRISPR/Cas9 genome editing to modify a short region of the mouse *Star* promoter (containing a subset of regulatory elements, including conserved CRE, C/EBP, AP1, and GATA motifs) that has been proposed to be critical for *Star* transcription. Analysis of the resultant mutant mice showed that this short promoter region is indeed required for maximal STAR mRNA and protein levels in the testis. Analysis also showed that both basal and hormone-activated testosterone production in mature mice was unaffected despite significant changes in *Star* expression. Our results therefore provide the first in vivo validation of regulatory sequences required for *Star* gene expression.

## 1. Introduction

Testosterone is the main sex hormone produced by the mature testis. It is essential for establishing biological maleness in typical XY males: development of the male urogenital tract during fetal development, acquisition of male secondary sex characteristics at puberty, and fertility in adults. Testosterone is synthesized and secreted by Leydig cells present in the testicular interstitium through the process of steroidogenesis. Steroidogenesis is the multistep conversion of cholesterol into steroid hormones via the sequential action of multiple proteins/enzymes. Steroidogenesis needs to be tightly regulated as too little or too much steroid hormone production can lead to incidences of differences of sex development (DSD) or pathologies such as congenital adrenal hyperplasia (CAH), osteopenia, and hormone-related cancers (reviewed in [1]). The regulation of testicular steroidogenesis is complex and involves many regulatory molecules and mechanisms such as luteinizing hormone (LH), its signaling pathways, and the factors that interpret these signals [1]. The latter include most notably transcription factors acting on the expression of genes that encode steroidogenic enzymes and other proteins involved in the biosynthesis of testosterone (reviewed in [2,3]).

Members of the GATA family of transcription factors are emerging as important regulators of steroidogenesis (reviewed in [4,5,6]). The GATA family is comprised of six factors (GATA1 to 6) that recognize and bind to the DNA motif (A/T)GATA(A/G) through their two conserved zinc finger domains. GATA factors are found in a broad array of tissues where they participate in cell differentiation, organogenesis, and the control of tissue-specific gene expression (reviewed in [4,7]). In both male and female gonads, GATA factors, especially GATA4 and/or GATA6, are crucial for the formation of the urogenital ridge, sex determination (gonad differentiation), fertility, and most likely steroidogenesis (reviewed in [4,7,8]). Insights into the GATA-dependence of steroidogenesis have come mainly from studies done in whole testis or immortalized Leydig cell lines. For example, GATA4 knockdown in either MA-10 or MLTC-1 Leydig cells represses the steroidogenic gene expression program and ultimately the production of sex steroid precursors [9,10]. In mice, loss of GATA4 and/or GATA6 function in the testis appears to block steroidogenic cell development and concomitant undermasculization of male embryos [11]. One of the testicular genes shown to be prominently affected by a modulation of GATA4/6 gene expression or activity is the gene encoding the steroidogenic acute regulatory protein (STAR).

Steroidogenic acute regulatory protein (STAR or STARD1) is a member of the larger START domain family, a group of transport proteins that share a common functional domain, the STAR-related lipid-transfer domain. In humans, 15 members (STARTD1-15) of the START domain family are known to transport cholesterol, ceramide, phosphatidylcholine, phosphatidylethanolamine (PE), and bile acids (reviewed in [12]). The first STARD protein discovered was the STAR or STARD1 protein which transports cholesterol from the outer to the inner mitochondrial membrane in steroidogenic cells of both the adrenals and gonads (reviewed in [13,14]). Cholesterol transport into the mitochondria is the rate-limiting step of steroidogenesis. STAR function in steroidogenesis was confirmed by both human disease (lipoid congenital adrenal hyperplasia/lipoid CAH) where the *STAR* gene is mutated [14,15], and in *Star* null mice which have a similar steroidogenic defect as seen in human lipoid CAH [16]. Being the rate-limiting step in steroidogenesis, STAR expression and activity is acutely regulated at both the transcriptional and post-transcriptional levels (reviewed in [13]).

The transcriptional control of the *STAR* gene has been intensely studied (reviewed in [13]). The proximal *STAR* promoter contains many tightly clustered regulatory motifs for the binding of different transcription factors that have been shown to modulate *STAR* promoter activity in vitro. These include motifs for the binding of NR5A factors (SF1/NR5A1 and LRH1/NR5A2), NR4A factors (NUR77/NGFI-B/NR4A1, NURR1/NR4A2, and NOR1/NR4A3), CCAAT/enhancer binding protein β (C/EBP β), CREB family factors (cAMP response element (CRE)-binding protein (CREB), CRE modulator (CREM), and sterol element binding protein (SREBP)), activator protein 1 (AP1), dosage-sensitive sex reversal, adrenal hypoplasia critical region, on chromosome X, gene 1 (DAX1), and Ying Yang 1 (YY1) (reviewed in [13]). As previously mentioned, GATA factors, mostly notably GATA4, are known to bind to the proximal *Star* promoter and enhance its transcription in vitro both basally and in response to acute hormonal stimulation [17,18]. The endogenous *Star* gene, however, has not yet been validated as a direct target for GATA binding or other transcription factors proposed to modulate its expression.

In this study, we report a novel mouse model created by CRISPR/Cas9 genome editing that modifies the mouse proximal *Star* promoter region to inactivate the critical GATA-binding motif as well as an adjacent 19-bp deletion that removes additional CRE, C/EBP, and AP1 binding motifs. Our results show that this short promoter region containing the GATA motif is required for endogenous *Star* expression in both fetal and postnatal mouse testes.

## 2. Results

### 2.1. The Integrity of a Short Promoter Region Harboring a Conserved GATA-Binding Motif Is Essential for Maximal Star Gene Expression in the Mouse Testis

**Figure 1:**
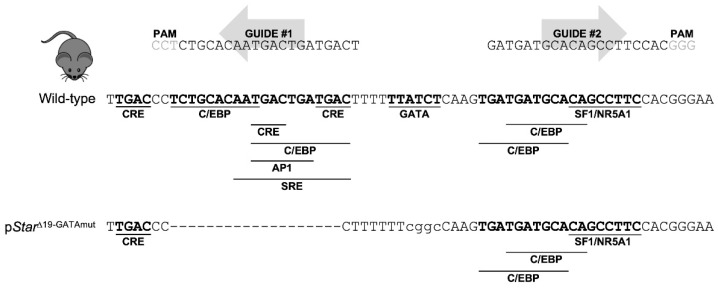
CRISPR/Cas9 genome editing strategy to target a conserved GATA regulatory element in the mouse *Star* promoter. The sgRNA and associated PAM sequences used are shown flanking the *Star* promoter GATA motif; regulatory motifs are underlined. Of the more than 100 founder mice screened, only 1 (named p*Star*^Δ19-GATAmut^) had a significant modification—a short 19-bp deletion generated by nonhomologous end-joining repair (NHEJ) and an adjacent mutated GATA motif introduced by the ssODN template during homology-directed repair (HDR). The 19-bp deletion removed CRE, C/EBP, AP1, and SRE binding motifs. Breeding of the p*Star*^Δ19-GATAmut^ founder with C57BL/6J mice confirmed transmission of the mutant allele.

Over the last two decades, the importance of GATA factors for the development and functioning of the mammalian gonads has been demonstrated through the analysis of GATA loss-of-function models in mice as well as the in vitro characterization of the GATA-dependence of the promoter regions of potential target genes (reviewed in [4,7,8]). The validation of these genes, however, as direct targets for GATA factors has been more of a challenge. CRISPR/Cas9 genome editing now offers the possibility of directly addressing this important question by allowing the precise targeting and modification of the genomic regions where GATA factors bind to their target genes. We used this strategy to define the anti-Müllerian hormone gene as a genuine target for GATA binding in Sertoli cells of the testis [19]. We now describe here the generation of a new mouse model using CRISPR/Cas9 editing to assess the importance of GATA binding for *Star* expression. As overviewed in Figure 1, the proximal mouse *Star* promoter contains a lone conserved GATA binding motif. Two sgRNAs were used in microinjections to maximize the chances of obtaining genome modifications that targeted the *Star* GATA motif. Less than 2% of founder mice (2 out of 105 births) were successfully targeted by the sgRNAs. Of these 2 founders, one was successfully repaired by the donor ssODN and contained the desired mutation of the GATA motif of the *Star* promoter. This same mouse also presented a 19-bp deletion created by nonhomologous end joining immediately upstream of the mutated GATA motif that also inactivated CRE, C/EBP, and AP1 binding motifs (Figure 1). A second founder mouse presented a longer 48-bp deletion that removed an SF1/NR5A1 motif in addition to those for CRE, AP1, and GATA. Both founders were viable and fertile. However, only the first founder (which we named p*Star*^Δ19-GATAmut^) transmitted the modified allele to its progeny. Mice heterozygous for the Δ19-GATA mutation were crossed to generate homozygotes; mice resulting from this cross were born at the expected Mendelian frequencies with a male/female ratio of 1:1. Both male and female p*Star*^Δ19-GATAmut^ heterozygous and homozygous mice were visibly undisguisable from wild-type (WT) littermates. Mature homozygous p*Star*^Δ19-GATAmut^ mice from both sexes were also fully fertile and showed no indication of adrenal insufficiency that is characteristic of *Star* null mice [16].

**Figure 2:**
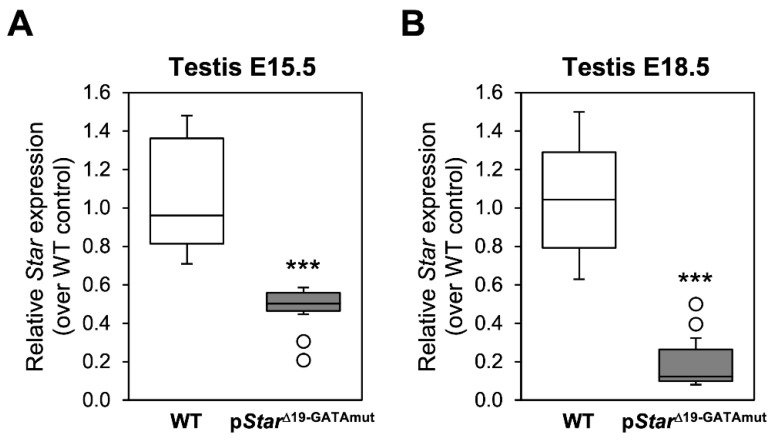
Comparison of *Star* gene expression in WT and p*Star*^Δ19-GATAmut^ fetal testis. *Star* expression was assessed by qPCR at (**A**) E15.5 and (**B**) E18.5. Data are reported as the value relative to age-matched WT mice (n = 6 to 11). Open circles are outliers. ***, significantly different from WT mice (*p* < 0.001).

**Figure 3:**
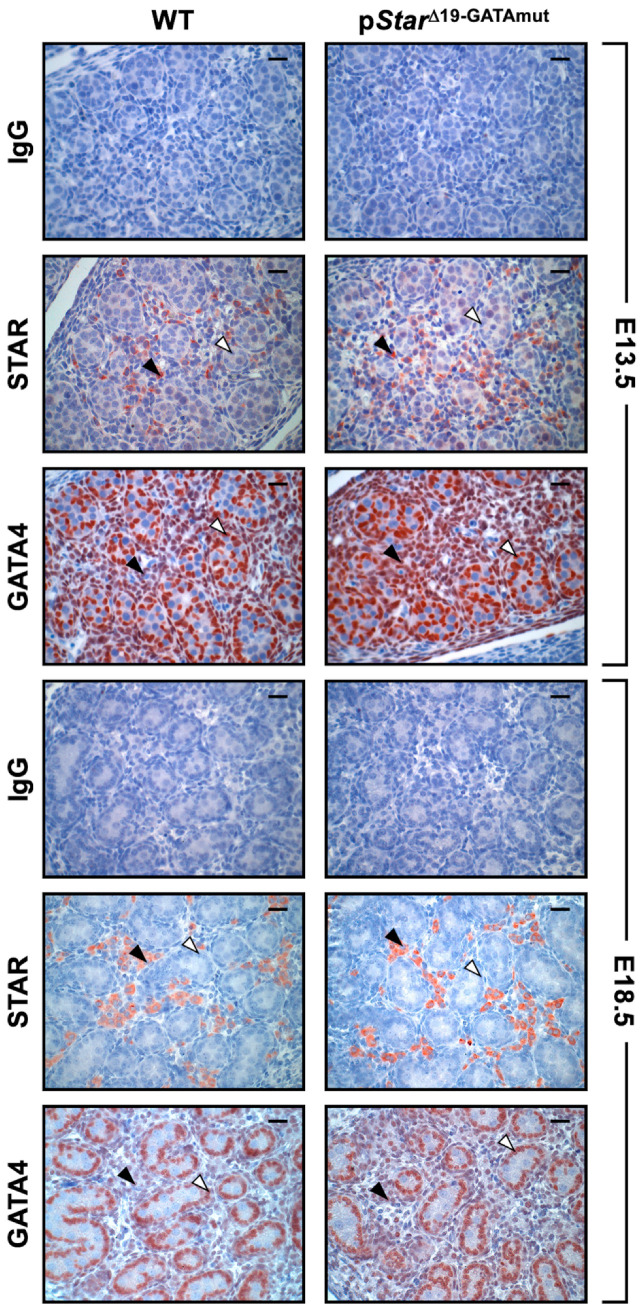
In situ detection of STAR and GATA4 protein in WT and p*Star*^Δ19-GATAmut^ fetal testes. Immunohistochemistry was performed on paraffin testis sections from both mouse genotypes at E13.5 and E18.5 using antibodies specific for STAR or GATA4; rabbit IgG was used as a negative control. Images were taken at 400× magnification; bar = 25 µm. Open arrowheads, GATA4-positive Sertoli cells; filled arrowheads, STAR-positive Leydig cells.

**Figure 4:**
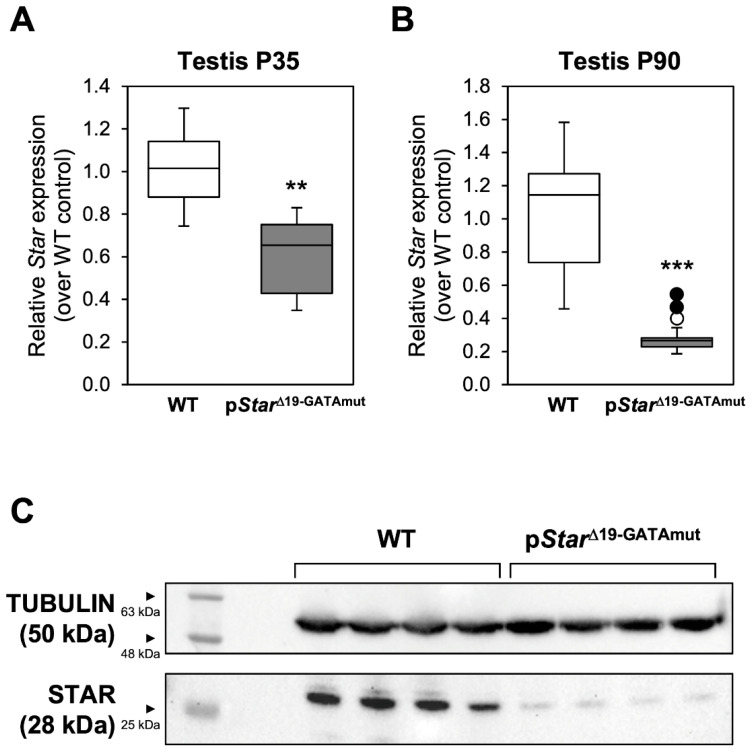
*Star* mRNA expression and STAR protein in postnatal testis of WT and p*Star*^Δ19-GATAmut^ mice. *Star* expression was assessed by qPCR in (**A**) juvenile P35 and (**B**) adult P90 testes. Data are reported as the value relative to age-matched WT mice (n = 6 to 10). The open circle is an outlier; filled circles are definitive outliers. Significantly different from WT: ** *p* < 0.01 for P35 testis and *** *p* < 0.001 for P90 testis. (**C**) Western blot detection of STAR protein levels in WT and p*Star*^Δ19-GATAmut^ adult mouse testis. TUBULIN was used as a loading control.

**Figure 5:**
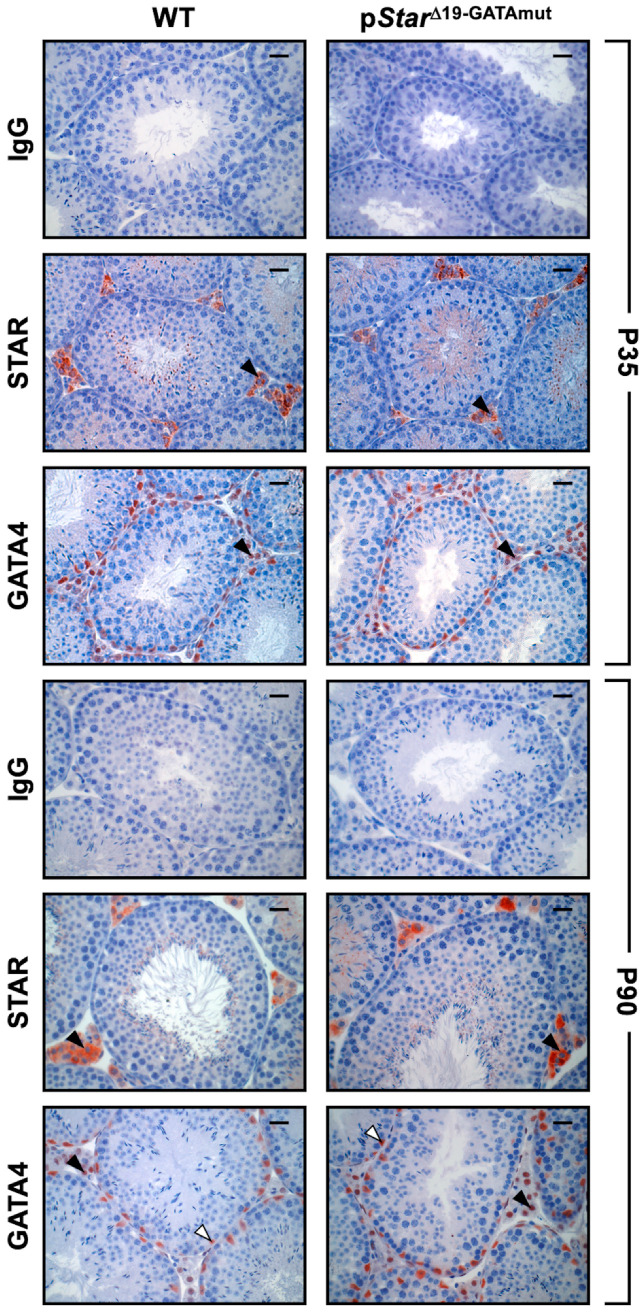
In situ detection of STAR and GATA4 protein in WT and p*Star*^Δ19-GATAmut^ postnatal testes. Immunohistochemistry was performed on paraffin testis sections obtained from both mouse genotypes at P35 and P90 using antibodies specific for STAR or GATA4; rabbit IgG was used as a negative control. Images were taken at 400× magnification; bar = 25 µm. Open arrowheads, GATA4-positive Sertoli cells; filled arrowheads, STAR-positive Leydig cells.

Despite exhibiting no overt phenotype, we examined whether *Star* gene expression was nonetheless affected in p*Star*^Δ19-GATAmut^ males. *Star* mRNA levels were first assessed in male gonads at embryonic day 15 (E15.5) and E18.5, time points that cover the critical masculinization programming window and the fetal testosterone surge in the mouse. *Star* mRNA levels in homozygous p*Star*^Δ19-GATAmut^ males were significantly reduced at both E15.5 (Figure 2A; 55% decrease) and E18.5 (Figure 2B; 79% decrease) when compared to testes from age-matched WT mice. We then performed immunohistochemistry on fetal testis sections to ascertain whether the reduction in *Star* mRNA levels translated to a similar decrease at the protein level (Figure 3). Beginning at E13.5, a time point when testis differentiation is complete and when testosterone production begins, there was no difference in the pattern of STAR protein (localization and level) between WT and p*Star*^Δ19-GATAmut^ mice. No change was also observed at E18.5 when the fetal testis is actively producing testosterone. We then examined testis *Star* expression and STAR protein in WT and mutant mice at later life stages (Figure 4): (1) just after puberty (P35) when Leydig cells are ramping up testosterone production and seminiferous tubules have engaged their first cycle of spermatogenesis, and (2) at adulthood (P90) when both testosterone levels and spermatogenic output are at their peak. As we observed for the fetal testis, *Star* mRNA levels were significantly lower in p*Star*^Δ19-GATAmut^ mice than in the WT counterparts—both in P35 juvenile adults (Figure 4A; 40% less) and P90 mature adults (Figure 4B; 75% less). Western blotting on whole extracts from adult testes also showed a consistent and marked reduction in STAR protein in p*Star*^Δ19-GATAmut^ mice when compared to age-matched WT control animals (Figure 4C). Immunohistochemistry, however, revealed no change in cellular localization of STAR protein between p*Star*^Δ19-GATAmut^ and WT testes at both postnatal ages (Figure 5).

### 2.2. Changes in Star mRNA and STAR Protein Levels in pStar^Δ^^19-GATAmut^ Mice Are Not Correlated with Alterations in Basal or Hormone-Induce Testosterone Production

**Figure 6:**
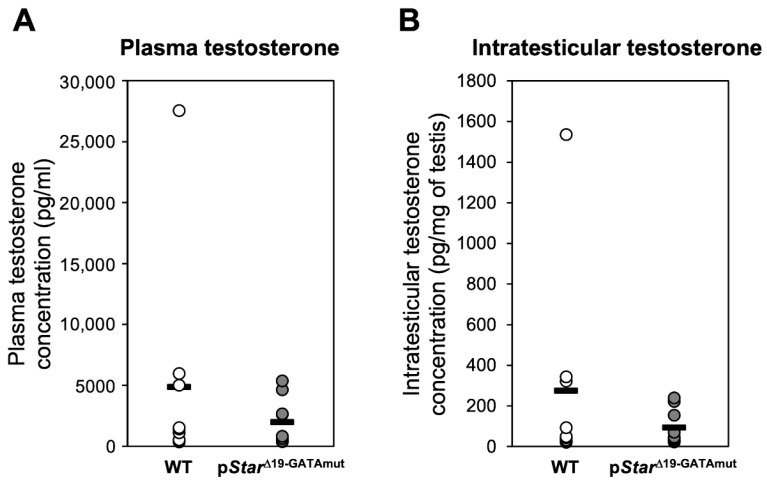
Testosterone levels in WT (open circles) and p*Star*^Δ19-GATAmut^ (filled circles) adult P90 male mice. (**A**) Plasma testosterone is expressed as ng/mL (n = 9). (**B**) Intratesticular testosterone is expressed as pg/mg testis (n = 9); bar = average. No significant difference was observed between WT and p*Star*^Δ19-GATAmut^ mice for both plasma and intratesticular testosterone levels.

**Figure 7:**
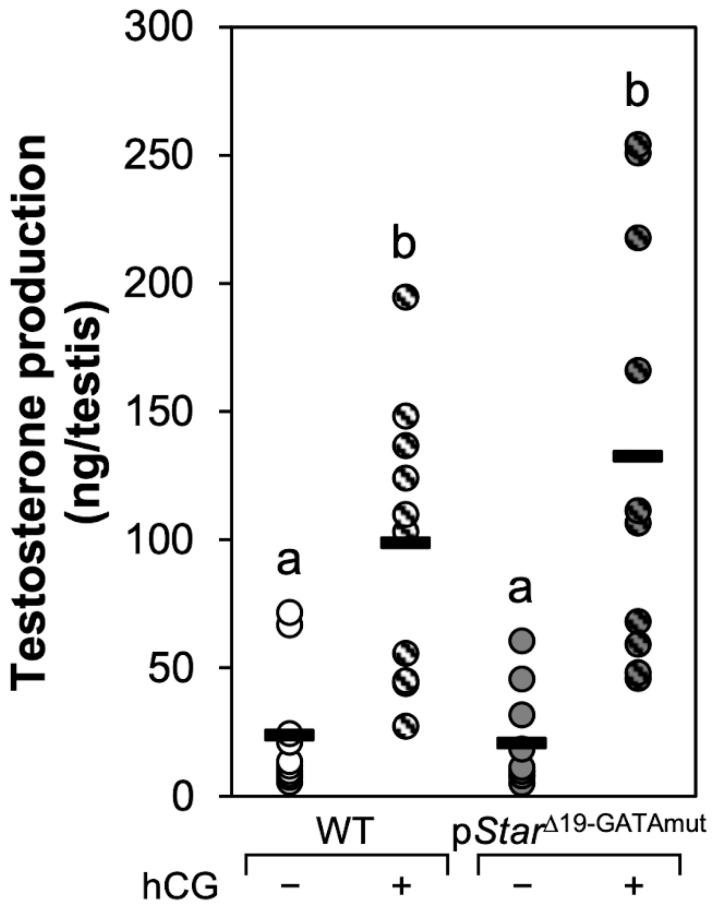
Ability of adult WT and p*Star*^Δ19-GATAmut^ testis explants to respond to hCG stimulation. Detunicated adult testis from WT or p*Star*^Δ19-GATAmut^ mice were placed in culture medium with antibiotics and treated with or without 1 IU/mL of hCG. After 4 h, culture media were also collected and assayed for testosterone by ELISA. Testosterone production is expressed as ng/testis (n = 10). Each individual sample is represented by a circle; bar = average. Open circles, WT in the absence of hCG; open hatched circles, WT treated with hCG; filled circles, p*Star*^Δ19-GATAmut^ in the absence of hCG; filled hatched circles, p*Star*^Δ19-GATAmut^ treated wikth hCG. Groups identified by different letters are significantly different, *p* < 0.05.

In steroidogenic cells, the transfer of cholesterol from the outer to the inner membrane of the mitochondria mediated by STAR is the rate-limiting step of steroid biosynthesis. Therefore, reduced *Star* mRNA and/or STAR protein levels observed in p*Star*^Δ19-GATAmut^ testes might compromise steroidogenesis and lead to a reduction in testosterone production. To test this hypothesis, we compared basal plasma and intratesticular testosterone levels in adult P90 WT and p*Star*^Δ19-GATAmut^ male mice (Figure 6). Although both basal plasma and intratesticular T were slightly reduced in p*Star*^Δ19-GATAmut^ males, the difference with WT mice was not statistically significant. Testicular *STAR* expression and activity are acutely regulated by the gonadotropin LH (reviewed in [13,20]). LH stimulation also induces steroidogenesis in the testis, which triggers numerous signaling pathways, which in turn can activate several transcription factors including GATA4 (reviewed in [2]). We therefore hypothesized that acute *Star* regulation might be impaired in testes of p*Star*^Δ19-GATAmut^ mice. Testis explants from both WT and p*Star*^Δ19-GATAmut^ adult P90 mice were placed in culture and exposed to a stimulating dose of hCG or left untreated. After 4 h in culture, we assessed the amount of testosterone released by the tissue ex vivo explants (Figure 7). Although testosterone production was induced after hCG treatment, there was no significant difference in hCG responsiveness between WT and p*Star*^Δ19-GATAmut^ testes.

## 3. Discussion

Steroidogenic acute regulatory protein (STAR), identified nearly three decades ago, is an essential cholesterol transporter in all steroidogenic tissues [21]. The delivery of cholesterol from the outer to the inner mitochondrial membrane mediated by STAR is the rate-limiting step in steroidogenesis [13]. As such, many studies have been devoted to understanding how *STAR* gene expression and protein activity are regulated in steroid producing tissues such as the adrenals and gonads. At the transcriptional level, the regulation of the *STAR* gene is complex, involving the interaction of numerous transcription factors to species-conserved binding motifs that are tightly clustered, and sometimes overlapping, located within the first few hundred base pairs upstream of the transcription initiation site (reviewed in [13]). Although many transcription factors have been shown to participate in *STAR* transcription across many species, to our knowledge, this evidence has been essentially limited to in vitro studies performed in cell lines or isolated steroidogenic tissues. No studies have yet probed the importance of these transcription factor binding sites for *Star* transcription in an in vivo whole animal context. In the present study, we have generated the first such mouse model (p*Star*^Δ19-GATAmut^), using CRISPR/Cas9 genome editing, to inactivate a short region of the mouse *Star* promoter containing a subset of these transcription factor binding sites. Analysis of the mutant mice confirmed that they are indeed essential for maximal expression of the endogenous *Star* gene but not basal or hormone-activated testosterone production.

The initial goal of our genome editing effort was to inactivate the lone GATA regulatory motif in the mouse *Star* promoter that has been long proposed to be critical for *Star* transcription [18]. However, even after screening more than 100 founder mice, we were unable to exclusively alter the *Star* GATA-binding motif. This was not totally unexpected given the low rate of homology directed repair (HDR) [22]. Despite this, we were successful in creating a mouse (p*Star*^Δ19-GATAmut^) with a short 19-bp deletion in tandem with a mutated GATA motif (see Figure 1). The deleted region contains six additional regulatory motifs—many of them overlapping—for various transcription factors, including members of the C/EBP and CREB families. The sequence covered by the Δ19-GATA mutation spans one of two critical regions that largely govern the positive modulation of *STAR* expression as documented through in vitro studies (reviewed in [20,23]). The analysis of our p*Star*^Δ19-GATAmut^ mice confirmed that this region is indeed important for maximal *Star* expression in fetal, peripubertal, and adult testes. However, we cannot at present formally attribute the decrease in *Star* expression to any one of these specific motifs since they were simultaneously disrupted in p*Star*^Δ19-GATAmut^ mice. Contrasting our in vivo findings with existing in vitro data do suggest that the CRE motifs are likely important—MA-10 Leydig cells transfected with CREB increases *Star* mRNA levels while mutation of at least the middle CRE motif (CRE2 in ref. [24]) reduces *Star* promoter activity by approximately 50% [24], a decrease comparable to what we observed for *Star* mRNA in p*Star*^Δ19-GATAmut^ testes. The proximal *Star* promoter also contains two C/EBP binding sites; one of these sites (site C2 identified in ref. [25]) was deleted in our p*Star*^Δ19-GATAmut^ mice. Mutation of this C/EBP motif significantly reduces *Star* promoter activity in MA-10 Leydig cells [25]. The same can be said for the GATA motif—the *Star* promoter is potently activated by GATA factors in cells, an effect that is completely lost when motif is mutated [18]. Considering the short length of the Δ19-GATAmut region as well as the very close proximity of the regulatory motifs present therein, it is reasonable to speculate that these motifs are not simultaneously bound by different transcription factors but rather are occupied by dynamic binding. Moreover, the proximity of the regulatory motifs further implies a physical closeness of the various DNA binding proteins that would permit the proteins to directly interact. Indeed, the existence of regulatory complexes among the different transcription factors has been well-documented. For example, C/EBP and CREB proteins interact with GATA4 to increase *Star* promoter activity, at least in vitro [17,26]. Therefore, we can now conclude that the integrity of this short region is critical not only for in vitro *Star* promoter activity but also for *Star* transcription in both fetal and postnatal testis in the mouse.

Although STAR protein is plentiful in Leydig cells, it was technically difficult for us to quantify it in early fetal testes. However, in adult testes, STAR was clearly less abundant in p*Star*^Δ19-GATAmut^ than in WT mice. Immunohistochemistry also showed that this was not accompanied by a change in cellular localization, suggesting that although less abundant, it should remain functional. Based on these observations, we expected that this would translate into reduced testosterone production either in plasma or locally within the testis itself. However, for both intratesticular and circulating (plasma) testosterone, levels in p*Star*^Δ19-GATAmut^ adult mice were not significantly different from WT. This was somewhat surprising knowing that genetic male *Star* null fetal mice have feminized external genitalia which is suggestive of a deficit in the production of androgen precursors from fetal Leydig cells [16]. Moreover, testosterone levels are 10 times lower at 8 weeks in serum from glucocorticoid-rescued *Star* null males when compared to age-matched controls [27]. This indicates that despite a 50–75% decrease in *Star* mRNA or STAR protein observed in p*Star*^Δ19-GATAmut^ testes, the amount of STAR remaining must be sufficient to allow for normal steroidogenesis. In steroidogenic tissues, *STAR* transcription is rapidly induced by the gonadotropin LH acting via a cAMP signaling pathway [28]. The short promoter region targeted in our p*Star*^Δ19-GATAmut^ mice also harbors regulatory elements known to confer LH responsiveness, including the GATA-binding motif (reviewed in [20]). Based on these facts, we surmised that a steroidogenic deficit might be more easily captured under conditions of hormonal stimulation. Yet again, we observed that ex vivo testis cultures from adult p*Star*^Δ19-GATAmut^ mice still responded to hCG stimulation when compared to WT controls, therefore reinforcing the notion that STAR protein, while diminished significantly in amount, was still adequate to elicit an induction of steroidogenesis when stimulated.

Taken together, our results provide new insights into the transcriptional regulation of the *Star* gene and highlight the power of CRISPR/Cas9 genome editing for validating the importance of proposed promoter regulatory sequences for both basal and acute hormone-stimulated gene regulation.

## 4. Materials and Methods

### 4.1. Animals

All mouse experiments were carried out in accordance with the Canadian Council of Animal Care guidelines for the care and manipulation of animals used in research. Protocols were approved by the Comité de Protection des Animaux de l’Université Laval, Québec, QC, Canada (protocol nos. 2019-149 and CHU-19-046).

### 4.2. CRISPR/Cas9 Generation of Mouse Star Promoter Mutants

A 100-bp genomic region of the proximal murine *Star* promoter spanning a single conserved GATA binding motif was searched using the CRISPOR Web tool (www.crispor.tefor.net, accessed on 15 June 2019) for potential single-guide RNA (sgRNA) sequences [29]. Guides were selected based on their low predicted off-target potential. A pX330-U6-Chimeric_BBCBh-hSpCas9 plasmid (no. 42230) purchased from Addgene (Cambridge, MA, USA) was used to generate SpCas9/chimeric sgRNA expression plasmids [30], as previously described [19]. A single-strand oligonucleotide (ssODN) was synthesized as a template for HDR of the double-strand breaks created by the sgRNAs. The ssODN contains a mutated GATA motif of the murine *Star* promoter flanked on each side by ~90-nucleotide-long homology arms. The oligonucleotides used as primers for creating the sgRNAs as well as the ssODN used as a donor for HDR are shown in Table 1 (the GATA motif is underlined and the mutated nucleotides are in lowercase). The SpCas9/chimeric sgRNA constructs were first validated in vitro and then microinjected along with the ssODN into fertilized C57BL/6J mouse eggs using the microinjection and transgenesis platform of the Institut de Recherches Cliniques de Montréal. A total of 105 founder mice were born and analyzed. Genomic DNA was isolated from tail tips collected from the founder mice using the HotSHOT method [31]. DNA screened for genomic rearrangements using genotyping primers (listed in Table 1) and Taq FroggaMix 2X master mix (FroggaBio, Concord, ON, Canada). PCR conditions were: initial denaturation for 3 min at 95 °C followed by 35 cycles of denaturation (30 s at 95 °C), annealing (30 s at 68 °C), and extension (30 s at 72 °C), and a final extension for 3 min at 72 °C. Amplicons were sequenced and analyzed using the Web tool TIDE (for tracking indels by decomposition) to evaluate the extent of Cas9-mediated rearrangements that occurred [32] and to identify the desired mutated or deleted alleles. Founder mice that targeted the GATA motif were crossed with C57BL/6J mice (stock no. 000664; The Jackson Laboratory, Bar Harbor, ME) to assess the transmission of the modified alleles. One founder mouse that possessed a combined GATA mutation and 19-bp deletion (named p*Star*^Δ19-GATAmut^) successfully transferred the modified *Star* promoter sequence to its offspring. Mice were backcrossed for a minimum of 5 generations with C57BL/6J mice to eliminate potential off-target effects. Heterozygous descendants were crossed to generate homozygous as well as wild-type (WT) control mice for experimentation as well as additional heterozygous mice that were used for colony maintenance.

### 4.3. Quantitative Real-Time RT-PCR

Testes were dissected from male mice at various developmental ages: embryonic day 15.5 (E15.5), E18.5, postnatal day 35 (P35, juvenile adult), and P90 (adult). Testes from E15.5, E18.5, and P35 testes were processed for total RNA extraction using TRI Reagent solution (Sigma-Aldrich Canada, Oakville, ON, Canada) in accordance with the manufacturer’s instructions. Testes from adult male mice were halved while frozen. One half was used for protein extraction (described below) and the other half was used for RNA extraction and intratesticular testosterone quantification (described below under *Hormone assay*). First-strand cDNA was synthesized from total RNA isolated from tissues using the iScript Advanced cDNA synthesis kit for quantitative real-time RT-PCR (qPCR; Bio-Rad Laboratories, Mississauga, ON, Canada). Assessment of gene expression by qPCR was done using a CFX96 plate thermal cycler and SsoAdvanced Universal SYBR Green Supermix from Bio-Rad Laboratories using their standard protocol. A panel of reference genes known for their stability in the mouse gonad [33], as well as typically used reference genes, were used for normalization. Primers for qPCR are listed in Table 2. Primer pairs were optimized beforehand for specificity and efficiency using a temperature gradient to identify the best annealing temperature and by performing a standard curve using a serial dilution of a pool of samples. PCR amplifications were run in duplicate under the following conditions: initial denaturation for 3 min at 95 °C followed by 40 cycles of denaturation (10 s at 95 °C), annealing (20 s at 62.6 °C), and extension (20 s at 72 °C) with a single acquisition of fluorescence level at the end of each extension step. Differences in mRNA levels between the mouse genotypes was determined using the ΔΔCq method [34].

### 4.4. Immunohistochemistry

Whole male embryos were collected at E13.5. For E18.5 fetuses as well as juvenile and adult mice, gonads were harvested and prepared for histological analysis. Immunohistochemical (IHC) staining was performed using the Rabbit Specific HRP/AEC IHC Detection Kit-Micro-polymer (ab236468; Abcam, Toronto, ON, Canada) following the manufacturer’s protocol. Tissue sections (4 µM) were deparaffinized and rehydrated in graded ethanols. Tissues were processed for antigen retrieval by treating them with citrate buffer (10 mM Sodium citrate, 0.05% Tween 20, pH 6.0) in a Decloaking Chamber™ NxGen (Biocare Medical, Pacheco, CA, USA) for 10 min at 110 °C. Sections were incubated overnight at 4 °C with primary antibodies for either a rabbit polyclonal anti-STAR IgG (Proteintech Cat# 12225-1-AP, RRID:AB_2115832) diluted 1:200 (1.7 μg/mL) in phosphate buffered saline (PBS) containing 1% bovine serum albumin (BSA) or a rabbit polyclonal anti-GATA4 IgG (Abcam Cat# ab84593, RRID:AB_10670538) diluted 1:500 (1.8 μg/mL) in PBS containing 1% BSA. Sections incubated with rabbit IgG isotype control (Invitrogen Cat # 02-6102, RRID:AB_2532938) diluted 1:2500 (2 μg/mL) in PBS containing 1% BSA were used as negative controls. All sections were counterstained with Harris Modified hematoxylin solution (Thermo Fisher Scientific, Nepean, ON, Canada) and mounted in MOWIOL (EMD Millipore, Gibbstown, NJ, USA). Slides were visualized with a Zeiss Axioscop II microscope (Carl Zeiss Canada, Toronto, ON, Canada) connected to a Spot RT Slider digital camera (Diagnostic Instruments, Sterling Heights, MI, USA). At least three animals per genotype were assessed.

### 4.5. Protein Extraction and Western Blot Analysis

For western blot analysis, half of a testis was homogenized while still frozen in ice-cold extraction buffer (50 mM Tris pH 7.4, 150 mM NaCl, 1 mM EDTA, 0.5% Igepal, 1 mM DTT, 0.5 mM phenylmethylsulfonyl fluoride (Sigma-Aldrich), and proteinase inhibitors (Sigma-Aldrich): 5 μg/mL aprotinin, 5 μg/mL leupeptin, and 5 μg/mL pepstatin. Homogenates were incubated on ice for 15 min after which the samples were briefly sonicated to break genomic DNA. Protein concentration was evaluated by Bradford assay [35], using Bio-Rad protein assay dye reagent concentrate (Bio-Rad Laboratories, Mississauga, ON, Canada) and BSA as protein standard. Aliquots (40 μg) of testicular homogenates were separated by SDS-PAGE and then electrotransferred to nitrocellulose membrane (Bio-Rad Laboratories, Mississauga, ON, Canada). Non-specific antibody binding was prevented by blocking for 1 h at RT using 5% non-fat dry milk in Tris-buffered saline (TBS: 20 mM Tris, 150 mM NaCl, pH 7.6) with 0.1% Tween 20 (TBS-T). Proteins were detected using commercially available primary antibodies for either a rabbit monoclonal anti-STAR IgG (Cell Signaling Technology Cat# 8449, RRID:AB_10889737) at a dilution of 1:5000 in 5% non-fat dry milk in TBS-T, a mouse monoclonal anti-α-tubulin IgG (used as a loading control, Sigma-Aldrich Cat# T5168, RRID:AB_477579) at a dilution of 1:10,000 in 5% non-fat dry milk in TBS-T. After washing in TBS-T, membranes were incubated with horseradish peroxidase-labeled secondary antibodies: goat anti-rabbit IgG (Vector Laboratories Cat# PI-1000, RRID:AB_2336198) diluted 1:5000 5% non-fat dry milk in TBS-T for STAR detection or horse anti-mouse IgG (Vector Laboratories Cat# PI-2000, RRID:AB_2336177) diluted 1:5000 in 5% non-fat dry milk in TBS-T for α-tubulin. After washing in TBS-T, membranes were finally incubated with Clarity Western ECL Substrate (Bio-Rad Laboratories) for 5 min. The chemiluminescent signal was detected on a ChemiDoc Imaging System (Bio-Rad Laboratories).

### 4.6. Hormone Assay

At the time of sacrifice, blood was drawn from adult mice by cardiac puncture. Blood was collected in EDTA-coated microtubes to prevent clotting, and plasma was isolated by centrifugation for 3 min at 2400 g and stored at −80 °C until further needed. For intratesticular testosterone quantification and RNA extraction, half a testis from each animal was homogenized while still frozen in cold PBS on ice and then centrifuged at 3000 rpm for 5 min at 4°C. Supernatant was collected and assayed for testosterone. The pellet was resuspended in TRI reagent to isolate total RNA for qPCR analysis (described above). For ex vivo stimulation of testosterone, testes were harvested and placed in 500 μL of DMEM containing 100 IU/mL penicillin and 100 μg/mL streptomycin. Testes were detunicated and treated with either 1 IU/mL of human chorionic gonadotropin (hCG, Sigma-Aldrich) or vehicle (H_2_O) and incubated for 4 h at 32 °C. After incubation, tissues were centrifuged for 5 min at 2400 g and the culture medium was collected. Testosterone quantification in plasma, testicular homogenates or culture medium was performed using an ELISA kit purchased from Cayman Chemical (Cayman Chemical, Ann Arbor, MI, USA, Cat# 582701, RRID:AB_2895148) following the instructions recommended by the manufacturer. To obtain readings within the range of the standard curve, samples were diluted in EIA buffer (provided by the kit manufacturer) as follows: plasma (1:20 and 1:50), testicular homogenates (1:100 and 1:200) and culture medium (1:200 and 1:500). Microplates were read using a Tecan Spark^®^ 10M multimode plate reader (Tecan, Morrisville, NC, USA). The assay has a cross reactivity of 100% for testosterone. Cross-reactivity is negligible for other sex steroids with the exception of 5α-dihydrotestosterone (DHT) at 27.4% (Cayman Chemical). However, since mouse intratesticular and plasma DHT levels are very low, this would not impact the testosterone measurements.

### 4.7. Statistical Analysis

Statistical analyses were done using JASP version 0.16.3 (https://jasp-stats.org, accessed on 10 October 2021; JASP Team (2022), Amsterdam, The Netherlands). Quantitative comparisons between wild-type and mutant mice (*Star* mRNA, plasma and intratesticular testosterone) were analyzed using a parametric Student’s *t*-test. Measurement of ex vivo testosterone production was analyzed by one-way ANOVA followed by Tukey multiple comparisons tests. For all statistical analyses, *p* < 0.05 was considered significant.

## Figures and Tables

**Table 1:** Oligonucleotide primers used for the generation and validation of genetically modified mice.

Utility	Forward Primer	Reverse Primer
Guide 1	5′-CACCGAGTCATCAGTCATTGTGCAG-3′	5′-AAACCTGCACAATGACTGATGACTC-3′
Guide 2	5′-CACCGATGATGCACAGCCTTCCAC-3′	5′-AAACGTGGAAGGCTGTGCATCATC-3′
ssODN	5′-AGTCTGCTCCCTCCCACCTTGGCCAGCACTGCAGGATGAGGCAATCATTCCATCCTTGACGCT CTGCACAATGACTGATGACTTTTTTcggcCAAGTGATGATGCACAGCCTTCCACGGCAAGCATTTA AGGCAGCGCACTTGATCTGCGCCACAGCTGCAGGACTCAGGACCTTGAAAGGCTC-3′	
Genotyping	5′-GCACCTCAGTTACTGGGCAT-3′	5′-ACACAGCTTGAACGTAGCGA-3′

In the ssODN sequence, the mutated GATA motif is underlined.

**Table 2:** Oligonucleotide primers used for qPCR.

Gene Product	Forward Primer	Reverse Primer
*Star*	5′-CAACTGGAAGCAACACTCTA-3′	5′-CCTTGACATTTGGGTTCCAC-3′
*Actb*	5′-CTGTCGAGTCGCGTCCACC-3′	5′-ATTCCCACCATCACACCCTGG-3′
*Gapdh*	5′-GTCGGTGTGAACGGATTTG-3′	5′-AAGATGGTGATGGGCTTCC-3′
*Polr2a*	5′-ATCAACAATCAGCTGCGGCG-3′	5′-GCCAGACTTCTGCATGGCAC-3′
*Ppia*	5′-CGCGTCTCCTTCGAGCTGTTTG-3′	5′-TGTAAAGTCACCACCCTGGCACAT-3′
*Rplp0*	5′-AGATTCGGGATATGCTGTTGGC-3′	5′-TCGGGTCCTAGACCAGTGTTC-3′
*Tuba1b*	5′-CGCCTTCTAACCCGTTGCTA-3′	5′-CCTCCCCCAATGGTCTTGTC-3′

Abbreviations: *Actb*, actin beta; *Gapdh*, glyceraldehyde 3-phosphate dehydrogenase; *Polr2a*, RNA polymerase II subunit A; *Ppia*, peptidylprolyl isomerase A; *Rplp0*, ribosomal protein lateral stalk subunit P0; *Tuba1b*, tubulin *α*1B.

## Data Availability

All data generated and analyzed during this study are included in this article.
